# Supplementary material for: Positive epistasis drives clavulanic acid resistance in double mutant libraries of BlaC β-lactamase
Source: Commun Biol. 2024 Feb 17;7:197. doi: 10.1038/s42003-024-05868-5 (PMC10874438; doi:10.1038/s42003-024-05868-5)
Supplement: Supplementary file 2 — Supplementary Information [file 42003_2024_5868_MOESM2_ESM.pdf]

Supplementary Information for

Positive epistasis drives clavulanic acid resistance in double mutant libraries of BlaC β-lactamase

Marko Radojković[1] and Marcellus Ubbink[1]

[1]Leiden Institute of Chemistry, Leiden University, Einsteinweg 55, 2333 CC Leiden, The Netherlands

*Corresponding author, e-mail: m.ubbink@chem.leidenuniv.nl

Supplementary Materials and Methods

Supplementary Figures

Supplementary References

SUPPLEMENTARY MATERIALS AND METHODS

Supplementary note 1. Construction of multi-site saturation libraries.

Four double-site saturation mutagenesis libraries were prepared using the nicking mutagenesis method [1], with modifications suggested by Kirby *et al.* [2]. A *BbvCI* digestion site was introduced in plasmid pUK21 carrying the wild-type *blaC* gene by a whole-plasmid synthesis approach (all primer sequences used in this work are listed in Table S1). The presence of the *BbvCI* site was confirmed by Sanger sequencing. Mutagenic primers carrying NNK/NNS degeneracy were phosphorylated by adding 7 µL of each 100 µM primer to the phosphorylation reaction mixture containing 3 µL T4 polynucleotide kinase buffer, 1 µL 10 mM ATP, 10 U T4 polynucleotide kinase, 18 µL of milliQ H_2O, and incubated for 1 h at 37 °C. Prior to use, 2 µL of primer targeting position 105 was mixed with 2 µL of primer targeting position 130, 132, 220 or 234 in a separate tube and diluted to a final volume of 40 µL with milliQ H_2O. The secondary primer was phosphorylated and diluted in the same way (1:20). ssDNA template was prepared in a reaction containing 0.76 pmol plasmid dsDNA, 2 µL 10× NEB CutSmart buffer, 10 U Nt.BbvCI, 10 U exonuclease III, 20 U exonuclease I, and 5 µL of milliQ H_2O to a final reaction volume of 20 µL in a PCR tube. The following thermal cycle program was used: 37 °C for 60 min, 80 °C for 20 min, hold at 12 °C. Next, 3.3 µL of each 1:20 diluted oligo mixture, 10 µL of 5X Phusion HF buffer and 16.7 µL of milliQ H_2O were added to each tube (final volume of 50 µL), and the oligos were annealed with the following program: 98 °C for 2 min, 55 °C for 5 min, and hold at 55 °C. While the reactions were held on the block, the following was added to each tube: 10 µL 5x Phusion HF buffer, 20 µL 50 mM DTT, 1 µL 50 mM NAD^+, 2 µL 10 mM dNTPs, 200 U Taq DNA ligase, 2 U Phusion high-fidelity DNA polymerase and 11 µL of milliQ H_2O (final volume of 100 µL). The tube content was mixed thoroughly by pipetting and the following thermocycling program was used: 72 °C for 10 min, 45 °C for 60 min, hold at 12 °C. Each reaction was then column purified using Monarch PCR & DNA cleanup kit (New England BioLabs) and eluted in 15 µL of milliQ H_2O. Next, for the second template degradation reaction, the following was added to each tube: 2 µL 10× NEB CutSmart buffer, 1 U Nb.BbvCI, 2 U exonuclease III, and 20 U exonuclease I (20 µL final volume). The following thermocycler program was used: 37 °C for 60 min, 80 °C for 20 min, hold at 12 °C. To synthesize the second (complementary) mutant strand, the following was added to each reaction: 20 µL 5X Phusion HF buffer, 20 µL 50 mM DTT, 1 µL 50 mM NAD+, 2 µL 10 mM dNTPs, 3.3 µL 1:20 diluted phosphorylated secondary primer, 200 U Taq DNA ligase, 2 U Phusion high-fidelity DNA polymerase and 27.7 µL of milliQ H_2O. The tube content was mixed thoroughly by pipetting and the following thermocycling program was used: 98 °C for 30 s, 55 °C for 45 s, 72 °C for 10 min, 45 °C for 20 min, and hold at 12 °C. To degrade methylated and hemimethylated wild-type DNA, 40 U DpnI was added to each reaction and the mixture was incubated at 37 °C for 1 h. The final reaction was column purified using Monarch PCR & DNA cleanup kit and eluted in 6 µL of H_2O. The entire volume was used to transform 50 µL of KA797 electrocompetent cells. The cells were plated on Corning square bioassay dishes (Sigma-Aldrich) containing LB-agar with 50 µg/mL kanamycin and incubated overnight at 37 °C. Next day, colonies from each plate were scraped with 10 mL of LB broth, vortexed, and 1 mL of each plasmid library was used for plasmid isolation using GeneJET Plasmid Miniprep Kit (Thermo Fischer Scientific).

Table S1. Primer sequences.

**BbvCI insertion primers**	
BbvCI_pUK21_F	TCACACCGCATACGTCACCTCAGCAAGCAACCATAGTACGC
BbvCI_pUK21_R	GCGTACTATGGTTGCTTGCTGAGGTGACGTATGCGGTGTGA
**Nicking mutagenesis primers**	
I105_NNS_F	GATTACCTATACCAGTGATGATATCCGTAGCNNSAGTCCGGTTGCACAGCAGCATGTTCAGAC
S130_NNS_F	GGTCAGCTGTGTGATGCAGCAATTCGTTATNNSGATGGCACCGCAGCCAATCTGCT
G132_NNK_F	GATGCAGCAATTCGTTATAGTGATNNKACCGCAGCCAATCTGCTGCTGGCC
R220_NNS_F	ACGTAATACCACCGGTGCCAAANNSATTCGTGCAGGTTTTCCGGCAGATTGGAAAGTTATT
K234_NNS_F	CCGGCAGATTGGAAAGTTATTGATNNSACCGGTACGGGTGATTATGGTCGTGCAAAT
secondary_R	CACAGCTTGTCTGTAAGCGGATGC
**Amplicon primers**	
MR_NGS_105_F	GATGTGTATAAGAGACAGTGATTACCTATACCAGTGATGATATCCG
MR_NGS_130+132_R	CGTGTGCTCTTCCGATCTCAGATCGGCCAGCAGCAGATTG
MR_NGS_220+234_R	CGTGTGCTCTTCCGATCTCATTTGCACGACCATAATCACC

SUPPLEMENTARRY FIGURES

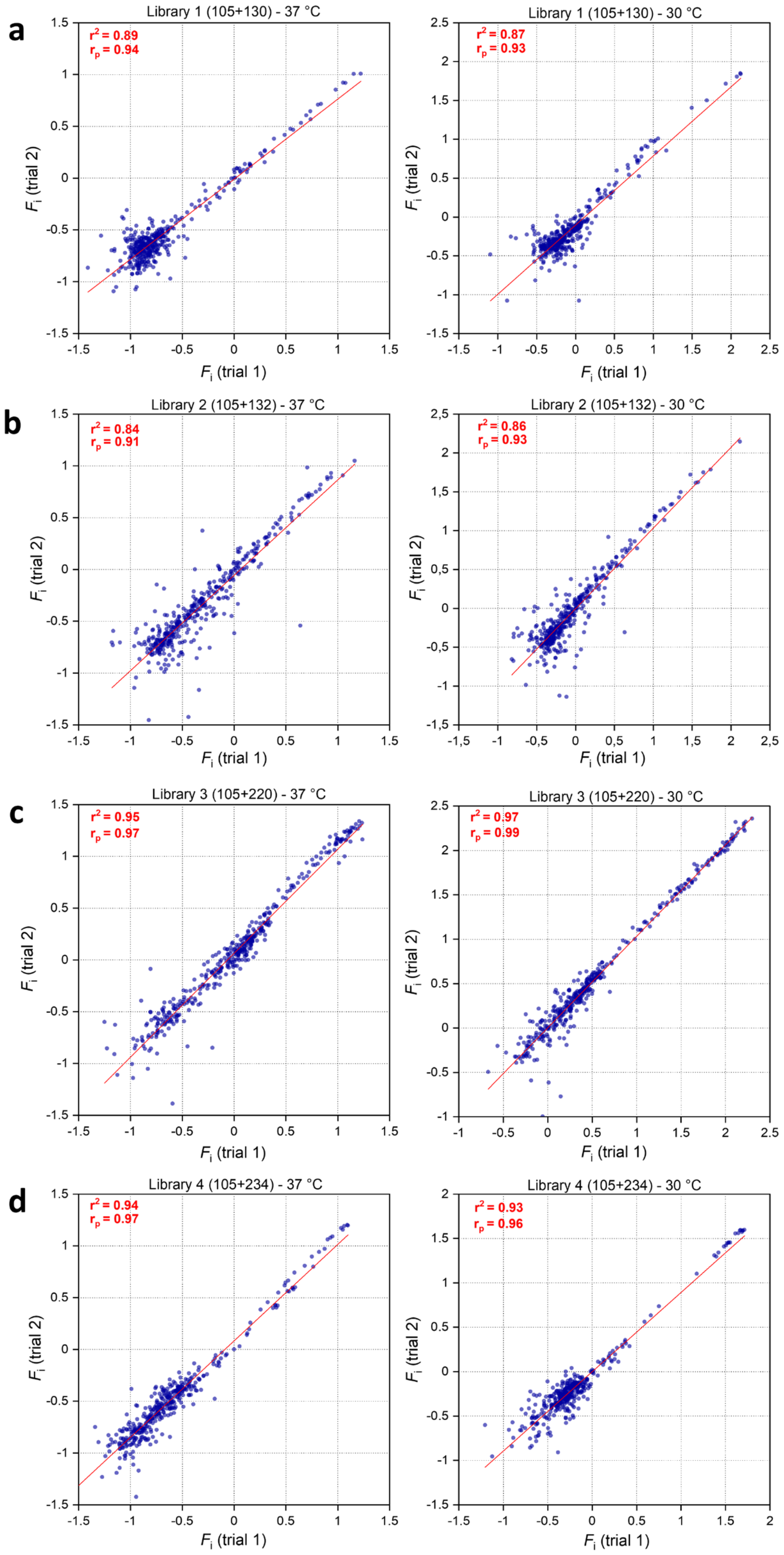

Figure S1. Data reproducibility. Shown is the correlation between fitness values of two independent experiments (trial 1 and 2) for all four libraries. (a) Library 1, (b) library 2, (c) library 3 and (d) library 4. Left, libraries selected at 37 °C; right, libraries selected at 30 °C. Red lines represent linear regression of which the correlation coefficient is given by r^2. The Pearson correlation coefficient is given by r_p.

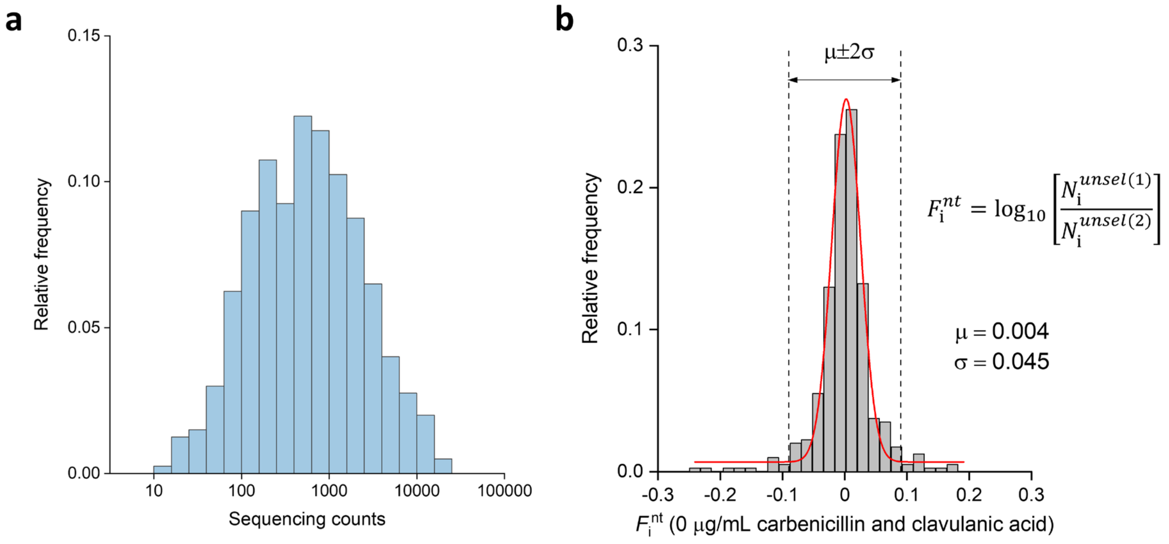

Figure S2. Experiment statistics. Shown as an example are the results for library 3 after growth at 37 °C. (a) Distribution of sequencing counts of the 400 alleles under conditions of no selection (0 µg/mL of carbenicillin and clavulanic acid). (b) The reproducibility of the sequencing counts under conditions of no selection (0 µg/mL of carbenicillin and clavulanic acid) of two independent, parallel experiments was used to set the limits for neutral effects. The neutral fitness effect (F_i^{nt}) was calculated from the ratio of normalized amino acid allele counts from the two parallel experiments (1) and (2), see equation. The red line indicates a fit to a Gaussian function; cutoffs for a statistically non-neutral effects on fitness are indicated as mean ± two × SD (µ = -0.004, σ = 0.045).

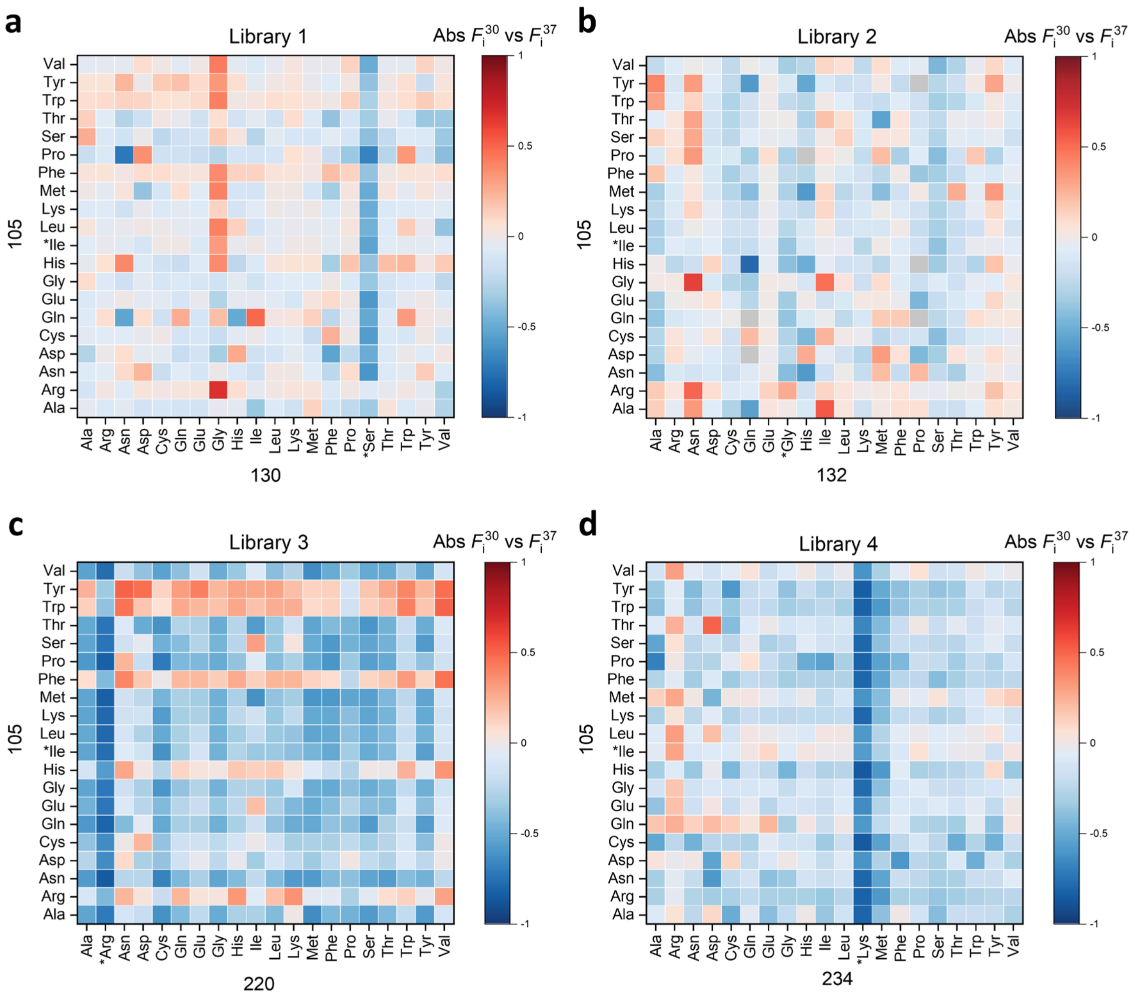

Figure S3. Difference in absolute fitness effects of all four library variants determined at 30 °C and 37 °C. (a-d) Libraries 1-4, positions 105/130 (a); 105/132 (b); 105/220 (c); 105/234 (d). Absolute fitness values determined at 37 °C were subtracted from absolute fitness values determined at 30 °C and difference values were plotted as heatmaps. Red indicates variants for which fitness is higher at 30 °C, and blue indicates variants for which fitness is higher at 37 °C. Variants for which the fitness effect could not be calculated are colored gray. The vertical axis depicts all possible amino acids on position 105 and the horizontal axis represents all possibilities at positions 130, 132, 220 or 234. Wild-type residues are marked with asterisk.

Table S2. Frequency of compensated double mutants (beneficial or neutral fitness), with one of the single mutants being deleterious.

	**Frequency (%)**	
	**37 °C**	**30 °C**
Library 1 (105+130)	2.8	22.7
Library 2 (105+132)	12.3	25.5
Library 3 (105+220)	22.7	17.2
Library 4 (105+234)	0.6	6.6
**Average**	**9.6**	**18.0**

SUPPLEMENTARY REFERENCES

1. Wrenbeck, E. E. *et al.* Plasmid-based one-pot saturation mutagenesis. *Nat. Methods* **13**, 928–930 (2016).

2. Kirby, M. B., Medina-Cucurella, A. V., Baumer, Z. T. & Whitehead, T. A. Optimization of multi-site nicking mutagenesis for generation of large, user-defined combinatorial libraries. *Protein Eng. Des. Sel.* **34**, 1–10 (2021).
