# Supplementary material for: Positive epistasis drives clavulanic acid resistance in double mutant libraries of BlaC β-lactamase
Source: Commun Biol. 2024 Feb 17;7:197. doi: 10.1038/s42003-024-05868-5 (PMC10874438; doi:10.1038/s42003-024-05868-5)
Supplement: Supplementary file 3 — Description of Supplementary Materials [file 42003_2024_5868_MOESM3_ESM.docx]

**Description of Additional Supplementary Files**

**File name:** Supplementary Data 1

**Description:** This file contains sequencing counts following quality filtering and calculated fitness and epistatic values. This is source data behind all Figures in the paper.
