# Supplementary material for: Positive epistasis drives clavulanic acid resistance in double mutant libraries of BlaC β-lactamase
Source: Commun Biol. 2024 Feb 17;7:197. doi: 10.1038/s42003-024-05868-5 (PMC10874438; doi:10.1038/s42003-024-05868-5)
Supplement: Supplementary file 5 — Reporting summary [file 42003_2024_5868_MOESM5_ESM.pdf]

nature portfolio

Corresponding author(s): Prof. Dr. Marcellus Ubbink

Last updated by author(s): Jan 16, 2024

Reporting Summary

Nature Portfolio wishes to improve the reproducibility of the work that we publish. This form provides structure for consistency and transparency in reporting. For further information on Nature Portfolio policies, see our Editorial Policies and the Editorial Policy Checklist.

Statistics

For all statistical analyses, confirm that the following items are present in the figure legend, table legend, main text, or Methods section.

n/a	Confirmed	
☐	☒	The exact sample size (n) for each experimental group/condition, given as a discrete number and unit of measurement
☐	☒	A statement on whether measurements were taken from distinct samples or whether the same sample was measured repeatedly
☒	☐	The statistical test(s) used AND whether they are one- or two-sided<br>*Only common tests should be described solely by name; describe more complex techniques in the Methods section.*
☒	☐	A description of all covariates tested
☒	☐	A description of any assumptions or corrections, such as tests of normality and adjustment for multiple comparisons
☒	☐	A full description of the statistical parameters including central tendency (e.g. means) or other basic estimates (e.g. regression coefficient) AND variation (e.g. standard deviation) or associated estimates of uncertainty (e.g. confidence intervals)
☒	☐	For null hypothesis testing, the test statistic (e.g. F, t, r) with confidence intervals, effect sizes, degrees of freedom and P value noted<br>*Give P values as exact values whenever suitable.*
☒	☐	For Bayesian analysis, information on the choice of priors and Markov chain Monte Carlo settings
☒	☐	For hierarchical and complex designs, identification of the appropriate level for tests and full reporting of outcomes
☐	☒	Estimates of effect sizes (e.g. Cohen's d, Pearson's r), indicating how they were calculated

Our web collection on statistics for biologists contains articles on many of the points above.

Software and code

Policy information about availability of computer code

Data collection	Illumina software (bcl2fastq v2.20) was used to acquire deep sequencing data in the form of FASTQ reads (by commercial partner).
Data analysis	Galaxy open web-based platform (https://usegalaxy.eu/) was used to analyze deep sequencing data.

For manuscripts utilizing custom algorithms or software that are central to the research but not yet described in published literature, software must be made available to editors and reviewers. We strongly encourage code deposition in a community repository (e.g. GitHub). See the Nature Portfolio guidelines for submitting code & software for further information.

Data

Policy information about availability of data

All manuscripts must include a data availability statement. This statement should provide the following information, where applicable:

- Accession codes, unique identifiers, or web links for publicly available datasets
- A description of any restrictions on data availability
- For clinical datasets or third party data, please ensure that the statement adheres to our policy

All data analysed during this study are included in this published article (and its supplementary files). Raw sequencing files are available through the NCBI SRA, under BioProject accession PRJNA1048706.

Research involving human participants, their data, or biological material

Policy information about studies with human participants or human data. See also policy information about sex, gender (identity/presentation), and sexual orientation and race, ethnicity and racism.

Reporting on sex and gender	Not applicable.
Reporting on race, ethnicity, or other socially relevant groupings	Not applicable.
Population characteristics	Not applicable.
Recruitment	Not applicable.
Ethics oversight	Not applicable.

Note that full information on the approval of the study protocol must also be provided in the manuscript.

Field-specific reporting

Please select the one below that is the best fit for your research. If you are not sure, read the appropriate sections before making your selection.

☐ Life sciences ☐ Behavioural & social sciences ☒ Ecological, evolutionary & environmental sciences

For a reference copy of the document with all sections, see nature.com/documents/nr-reporting-summary-flat.pdf

Ecological, evolutionary & environmental sciences study design

All studies must disclose on these points even when the disclosure is negative.

Study description	Site-saturation mutagenesis was used to generate all possible amino acid variants between four residue pairs (105+130, 105+132, 105+220, 105+234). Resulting four libraries were grown in liquid media containing 1 ug/mL clavulanic acid+ 100 ug/mL carbenicillin or without any antibiotic and inhibitor, at two different temperatures 37 and 30 °C, in duplicate.
Research sample	Four double site-saturation libraries of blaC gene on pUK21 plasmid.
Sampling strategy	All four libraries were individually generated by nicking mutagenesis.
Data collection	After growing each library under each condition and temperature (selected/unselcted; 30 or 37 °C), plasmid libraries were harvested and used as templates to generate amplicons for deep sequencing.
Timing and spatial scale	Each mutant library was grown 2.5 hours at 37 °C and 4 hours at 30 °C, followed by 16 hours of overnight growth without selection after which plasmid libraries were harvested.
Data exclusions	FASTQ reads that didn't pass quality and length filters were not used for further analysis.
Reproducibility	Simple linear and Pearson correlation between two sets of replicates was tested to ensure reproducibility.
Randomization	Not applicable, as the study aims to test a comprehensive sets of mutations.
Blinding	Not applicable, as the study does not rely on comparing effects of different treatments in a way that would benefit from blinding.

Did the study involve field work? ☐ Yes ☒ No

Reporting for specific materials, systems and methods

We require information from authors about some types of materials, experimental systems and methods used in many studies. Here, indicate whether each material, system or method listed is relevant to your study. If you are not sure if a list item applies to your research, read the appropriate section before selecting a response.

Materials & experimental systems

n/a	Involved in the study
☒	☐ Antibodies
☒	☐ Eukaryotic cell lines
☒	☐ Palaeontology and archaeology
☒	☐ Animals and other organisms
☒	☐ Clinical data
☒	☐ Dual use research of concern
☒	☐ Plants

Methods

n/a	Involved in the study
☒	☐ ChIP-seq
☒	☐ Flow cytometry
☒	☐ MRI-based neuroimaging

Plants

Seed stocks

Not applicable.

Novel plant genotypes

Not applicable.

Authentication

Not applicable.
